# Coordinated Turning Behaviour of Loitering Honeybees

**DOI:** 10.1038/s41598-018-35307-5

**Published:** 2018-11-16

**Authors:** Mandiyam Y. Mahadeeswara, Mandyam V. Srinivasan

**Affiliations:** 1 Queensland Brain Institute, University of Queensland, St Lucia, QLD Australia; 2 School of Information Technology and Electrical Engineering, University of Queensland, St Lucia, QLD Australia

**Keywords:** Motor control, Animal behaviour

## Abstract

Turning during flight is a complex behaviour that requires coordination to ensure that the resulting centrifugal force is never large enough to disrupt the intended turning trajectory. The centrifugal force during a turn increases with the curvature (sharpness) of the turn, as well as the speed of flight. Consequently, sharp turns would require lower flight speeds, in order to limit the centrifugal force to a manageable level and prevent unwanted sideslips. We have video-filmed honeybees flying near a hive entrance when the entrance is temporarily blocked. A 3D reconstruction and analysis of the flight trajectories executed during this loitering behaviour reveals that sharper turns are indeed executed at lower speeds. During a turn, the flight speed is matched to the curvature, moment to moment, in such a way as to maintain the centrifugal force at an approximately constant, low level of about 30% of the body weight, irrespective of the instantaneous speed or curvature of the turn. This ensures that turns are well coordinated, with few or no sideslips - as it is evident from analysis of other properties of the flight trajectories.

## Introduction

It is a common experience that driving too fast around a corner can cause a car to skid or roll over; a passenger standing in a bus can tip over if the bus makes a turn at high speed; or an aircraft attempting to make a very tight turn can experience a sideslip. The reason is that the act of turning while simultaneously moving forward creates a centrifugal force that is directed away from the centre of curvature of the turn. Newtonian mechanics dictates that the magnitude of the centrifugal force is proportional to (a) the curvature (the reciprocal of the radius of the turn), and (b) the square of the speed^1^. Hence, the sharper the turn, and the higher the speed, the greater the danger of losing control. Clearly, therefore, it makes sense to reduce one’s speed before commencing a turn, and to ensure that sharper turns are executed at a slower speed, in order to limit the centrifugal force to a safe and manageable value. This behaviour is adopted not only by car drivers, motorcyclists, bicyclists and runners, but also by several terrestrial and flying animals. Qualitative evidence to support such behaviour has been documented in race horses^2^, quolls^3^, houseflies^4^, fruitflies^5^, and bats^6^. However, a quantitative analysis of the relationship between flight speed and curvature, and the implications for the resulting centrifugal force that is experienced during turns, has not yet been explored in any animal.

Fruitflies (*Drosophila*) flying in a contained environment display segments of straight flight, interspersed with saccadic turns^7,8^. These turns are executed by performing a pitch and a roll of the body axis, which together induce a rapid change in the direction of flight. Visually evoked escape maneuvers of fruit flies also include sharp turns^9^, which are much faster than the stereotyped body saccades. While these turns enable rapid, aggressive changes of flight direction, they are inevitably associated with sideslips arising from the high centrifugal force. It is of interest to enquire whether flying insects are also capable of performing turns that are coordinated in such a way as to prevent sideslips - for example, during loitering flight. In our study, we induce bees to loiter in front of a beehive by blocking the entrance to the hive, which causes returning foragers to cruise in the vicinity of the hive entrance while they await entry. The behaviour of the bees in this ‘bee cloud’ is filmed using stereo video cameras and reconstructed in 3D to analyse their turning characteristics. The results reveal that loitering bees perform turns that are fully coordinated, and free of sideslips.

## Materials and Methods

**Figure 1:**
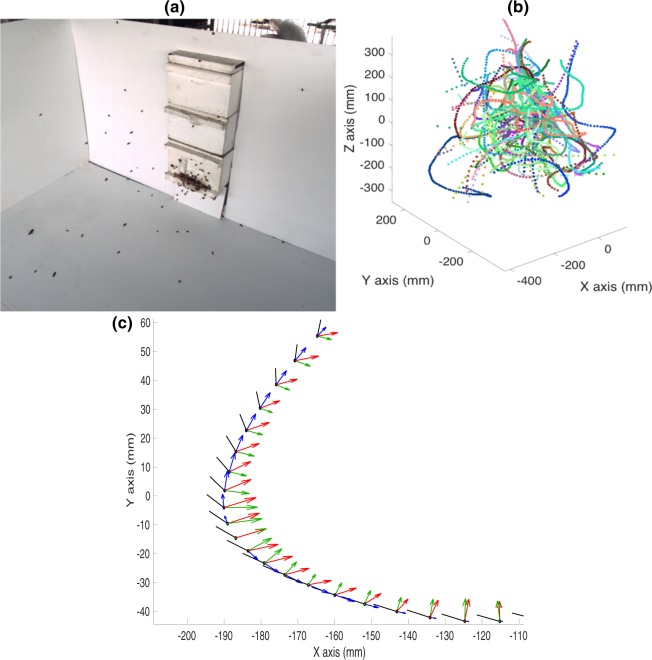
(**a**) Perspective view of the bee cloud. (**b**) Reconstructed 3D trajectories of 66 bees. (**c**) Successive head positions (dark circles) and body orientations (dark lines) of a bee (Bee 2), along with the acceleration and its vector components during a turn. The red, blue, and green arrows represent the total, tangential and centripetal acceleration vectors respectively.

A non-captive honeybee colony (*Apis Mellifera*) was maintained on a semi-outdoor terrace on the rooftop of a building on the campus of the University of Queensland (St. Lucia). The bees were allowed to forage freely from the surrounding vegetation, without any restrictions. The experiment was commenced by temporarily blocking the hive entrance with a wooden strip (Fig. 1a). The returning foraging bees were thus temporarily denied entry into the hive but flew near the vicinity of the hive entrance, making multiple attempts to gain access. The resulting ‘bee cloud’ was filmed using two synchronized digital cameras (Redlake), configured to obtain stereo data. The cameras recorded video at 60 fps with 500 × 500 pixel resolution.

Before commencing the experiment, stereo camera calibration was performed to obtain the cameras’ intrinsic and extrinsic parameters. The video streams acquired by the two cameras were subsequently analysed by digitising the bee’s head and tail positions manually in each frame, to obtain the bee’s position coordinates in each view. A triangulation routine was executed to obtain the three-dimensional positional coordinates of each bee. The 3D coordinates of a bee were computable only when it was within the FOV of both cameras. The recording duration was 5.8 seconds (349 frames). The frames in the video footage carried varying numbers of bees, as individual bees entered or departed from the fields of view (FOV) of the two cameras. The method used to compute the kinematic parameters of the flight were based on vector calculus^1^. The magnitudes of the tangential and normal accelerations at any time instant ‘t’ can be computed as

1 $${a}_{T}=\frac{r^{\prime} ({\boldsymbol{t}}).r^{\prime\prime} ({\boldsymbol{t}})}{\Vert r^{\prime} ({\boldsymbol{t}})\Vert }$$

2 $${a}_{N}=\frac{\Vert z^{\prime} ({\boldsymbol{t}})\times r^{\prime\prime} ({\boldsymbol{t}})\Vert }{\Vert r^{\prime} ({\boldsymbol{t}})\Vert }$$

where ${a}_{T}\,\triangleq$ tangential acceleration (TA) magnitude; ${a}_{N}\,\triangleq$ normal or centripetal acceleration (CA) magnitude; ${\boldsymbol{r}}({\boldsymbol{t}})\,\triangleq$ position vector; $r^{\prime} ({\boldsymbol{t}})\,\triangleq$ velocity vector and $r^{\prime\prime} ({\boldsymbol{t}})\,\triangleq$ total acceleration vector as function of time.

Mathematically, the curvature can be expressed as the rate of change of the unit tangent vector at a point. Using this concept, one can compute the magnitude of the curvature as function of time using the following vector algebra:

3 $$curvature\,k(t)=\frac{\Vert {\boldsymbol{r}}^{\prime} ({\boldsymbol{t}})\times {\boldsymbol{r}}^{\prime\prime} ({\boldsymbol{t}})\Vert }{{\Vert {\boldsymbol{r}}^{\prime} ({\boldsymbol{t}})\Vert }^{3}}$$

The radius of curvature (ρ) is expressed as the reciprocal of the curvature:

4 $$\rho (t)=\frac{1}{k(t)}$$

The raw data was pre-processed as follows: (i) A 5-point moving average filter was used to smooth the 3D position data; (ii) A central differencing method was used to compute a bias-free estimate of the gradient of the position vectors to compute the velocity vector, and subsequently the acceleration vector. No further filtering of these vectors was found to be necessary.

## Results

The bee cloud data contains 3D position coordinates of a total of 66 bees. Figure 1a shows a perspective view of the bee cloud at a particular instant of time. Figure 1b shows the reconstructed 3D trajectories of the 66 bees, where each colour represents the trajectory of an individual bee. We used techniques of vector calculus to examine the flight characteristics of bees maneuvering in the cloud, by computing the following parameters:

- The speed of each bee in the cloud, and its variation as a function of time;
- The acceleration of each bee in the cloud, and its tangential and centripetal components, and the variation of these parameters as a function of time;
- The curvature and radius of curvature (ROC) of the flight trajectory, and its variation with time.

**Figure 2:**
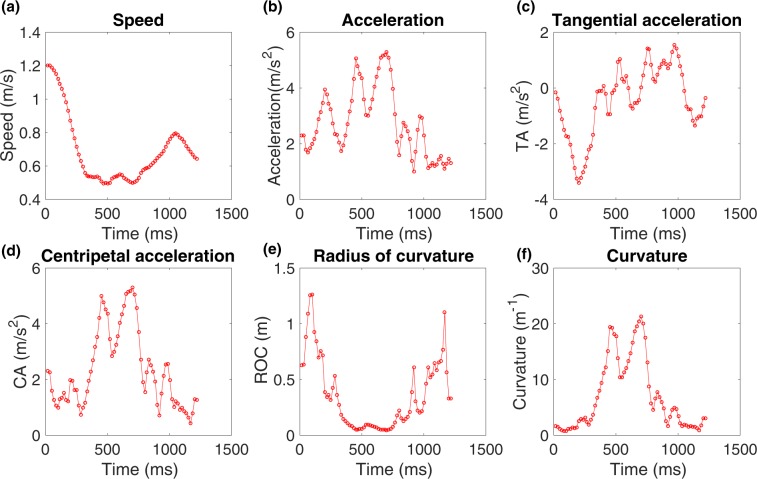
Variation of (**a**) speed, (**b**) acceleration, (**c**) tangential acceleration, (**d**) centripetal acceleration, (**e**) radius of curvature and (**f**) curvature of the trajectory of an individual bee (Bee 57) in the cloud.

A flight segment illustrating the successive head positions and body orientations of a bee (Bee 2) during a turn is shown in Fig. 1c. This figure includes vector representations of the acceleration, and of its tangential and centripetal components. The variation of each of the above parameters as a function of time is shown in Fig. 2 for a longer turning segment from a different bee (Bee 57).

### General relationship between instantaneous speed, curvature and centripetal acceleration

In general, the speed of a bee varies continuously through its flight path, as shown in Fig. 2a for an individual bee. The mean speed of this particular bee is 0.68 m/s, measured over its entire flight. Certain bees exhibited high speeds, despite flying in close proximity to other bees. For example, one individual reached a top speed of 2.61 m/s, while flying amidst 33 other bees in the cloud.

**Figure 3:**
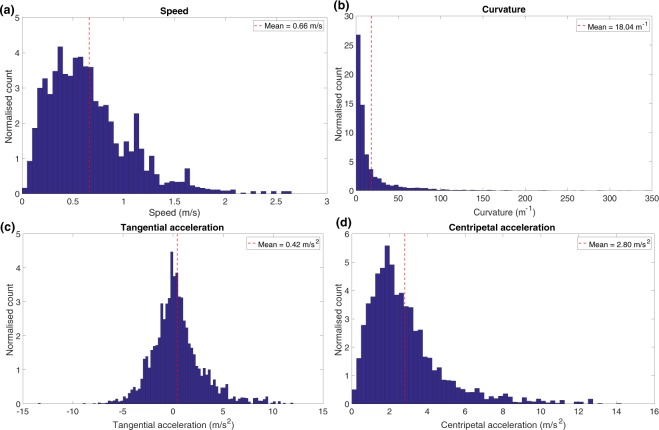
Average histogram of (**a**) speeds, (**b**) curvature magnitudes, (**c**) tangential acceleration and (**d**) centripetal acceleration magnitudes.

The average speed, measured over all bees in the cloud, was found to be 0.66 m/sec and the curvature of the trajectories displayed an average magnitude of 18 m^−1^. Average histograms of the speed and the curvature magnitudes of the trajectories of all 66 bees are shown in Fig. 3a,b, respectively. These histograms were obtained by computing an area-normalized histogram for each bee, and then averaging the results across the 66 bees.

The averaged histograms reveal a large variation in speed (ranging from 0 to 2.61 m/s; Fig. 3a), as well as curvature magnitude (ranging from 0 to ~300 m^−1^; Fig. 3b). However, these histograms include the variations across all bees, which have different mean speeds and mean curvature magnitudes.

A more representative measure of the average variability of speed and curvature within the trajectory of an individual bee is conveyed by the coefficient of variation (CV). This displays a value of 0.32 for speed, and 1.5 for curvature magnitude, when computed separately for each bee, and then averaged across all the bees.

Next, we calculated the tangential and centripetal components of the acceleration and plotted their variation as a function of time (Fig. 2c,d). The normalised histograms of tangential acceleration and the magnitude of the centripetal acceleration are shown in Fig. 3c,d respectively. The histogram of tangential acceleration clearly reveals that the flight contains acceleration and deceleration components, distributed approximately symmetrically about a value of zero (which corresponds to a constant tangential speed). The mean tangential acceleration, averaged across all bees, is 0.42 m/s^2^ (Fig. 3c), which is not significantly different from zero (*p* = 0.07; two tailed t-test). The mean standard deviation of the tangential acceleration is 2.0 m/s^2^. For many bees, the mean value of the tangential acceleration measured over the entire flight is very close to zero. Consequently, the CV of the tangential acceleration can become very large, approaching infinity, and not provide a useful measure of the variability of the tangential acceleration. A more useful measure is the CV of the *magnitude* of the tangential acceleration, which has a mean value of 1.86 m/s^2^, and a mean CV of 0.75, when computed separately for each bee, and then averaged across all bees. The relatively high CV value is likely due to the large variations in the magnitude of the tangential acceleration, which is maximal at the beginning and end of a turn, and zero near the middle.

The magnitude of the centripetal acceleration, averaged over each bee’s entire flight, and across all bees, has a mean value of 2.80 m/s^2^ (Fig. 3d), which is significantly different from zero (*p* = 2.6 × 10^−25^; two tailed t-test). This is obviously consistent with the fact that the bees are not always flying in straight lines, and that turns constitute a significant proportion of their flight trajectories. The mean CV of the magnitude of the centripetal acceleration is 0.51. Interestingly, the relatively low CV of the centripetal acceleration, compared to the CV for the tangential acceleration, raises the possibility that the centripetal acceleration may be regulated or restricted to particular limits while executing turns. This is explored in greater detail in the following section.

### Analysis of turning flights

We began by computing the overall mean speed of all bees during straight and turning segments.

Points along the flight trajectories at which the curvature magnitude was greater than 250 were not included in the plots, because such measurements could be dominated by the errors in the image digitization process. Points at which the curvature magnitude was lower than 5 were considered to represent flight in an approximately straight line. These curvature limits were used to select the turning parts of the flight trajectory, and exclude segments that corresponded to straight flight or very sharp turns.

The mean speed was 0.83 m/s (s.d. = 0.15 m/s) during straight flights and 0.49 m/s (s.d. = 0.12 m/s) during turning flights. These speeds are significantly different (*p* = 2.88 × 10^−08^; paired sample t-test), indicating that the bees fly at a significantly slower speed when they are executing turns.

**Figure 4:**
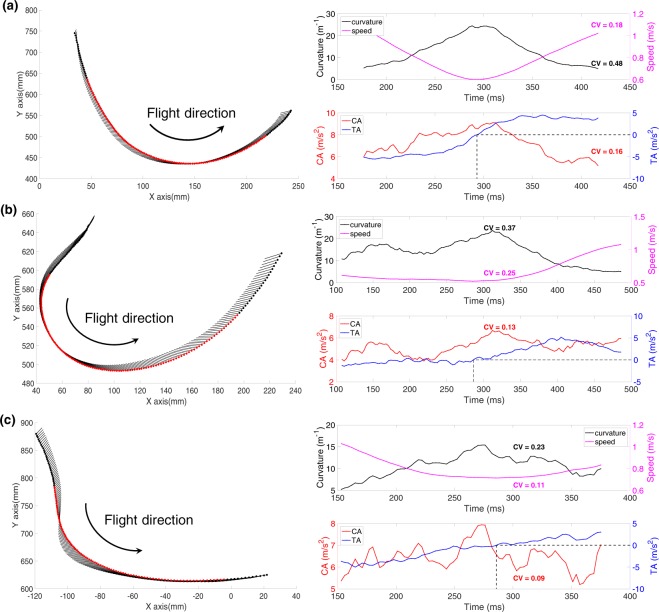
Variation of curvature, speed, centripetal and tangential acceleration during turning segments of three individual bees, shown in (**a**–**c**). In each subfigure the left hand panel shows the turning segment, with the red symbols defining the duration of the turning segment that was analysed. The upper right hand panel shows the variation of curvature (left ordinate) and speed (right ordinate) as a function of time (abscissa). The lower right hand panel shows the variation of centripetal acceleration (left ordinate) and tangential acceleration  (right ordinate) as a function of time. The dashed black lines show the zero-crossing point of the tangential acceleration. The numbers next to the curves show the CVs of the curvature, speed and centripetal acceleration. These flights were recorded at 335fps in order to visualise the variations of the turning parameters with a higher temporal resolution. Here, we applied a 29-point moving average filter to smooth the 3D position data, and subsequently a 11-point moving average filter to smooth out higher-level noise arising from computation of the second order derivatives.

We were interested to examine how the variables of speed, centripetal acceleration, tangential acceleration, and curvature, discussed in the previous section, vary during turning segments. By imposing a curvature threshold of 5 m^−1^–250 m^−1^ (ROC equivalent of 0.004 m–0.20 m) on the curvature data, we were able to extract the turning segments from the complete flight trajectory. We then estimated the temporal variation of curvature, speed, centripetal acceleration and tangential acceleration during these turning segments. Examples of this analysis for 3 different bees are shown in Fig. 4. These flights were recorded at 335fps in order to visualise the variations of the turning parameters with a higher temporal resolution. In each case, the magnitude of the curvature (dark curve, upper right-hand panels in Fig. 4a–c) is low at the beginning of the turn, reaches a maximum value at the middle of the turn, and then declines toward zero as the turn is completed. The flight speed, on the other hand, (magenta curve, upper right-hand panels in Fig. 4a–c) varies in the opposite sense. Each bee gradually decreases its speed while entering the turn, reaching a minimum speed close to the point of maximum curvature, and then subsequently speeds up. This behaviour is confirmed by the plots in the lower right-hand panels in Fig. 4a–c, which show that the tangential acceleration (blue curve) is negative during the first half of the turn and positive during the second half, indicating that the bee is decelerating while entering the turn and accelerating while exiting it. Thus, the tangential component of acceleration varies dramatically during the flight - even changing its polarity halfway through the turn.

On the other hand, the magnitude of the centripetal component of the acceleration is more or less constant throughout the turn, as illustrated by the red curve in the lower right-hand panels of Fig. 4a–c.

The CVs of the centripetal acceleration maintained by these three bees displayed relatively low values of 0.16, 0.13 and 0.09, respectively, as shown in Fig. 4, indicating that the centripetal acceleration remains more or less constant (relative to its mean value) during the turn. On the other hand, the variations of the curvature (CV = 0.48, 0.37, 0.23) and speed (CV = 0.18, 0.25, 0.11) are higher.

The relative constancy of the centripetal acceleration throughout the course of the turn suggests that bees may be orchestrating turns by varying the flight speed and the curvature, moment for moment, in such a way that the centripetal acceleration is held constant during the course of the turn.

Do bees really hold the CA constant during turns? To test this hypothesis, we examined the predictions of a constant-CA model as follows. We may write the centripetal acceleration as:

5 $$centripetal\,acceleration=\frac{{v}^{2}}{\rho }$$

where ‘*ρ*’ is the instantaneous radius of curvature of the trajectory, and ‘*v’* is the instantaneous bee speed.

If the centripetal acceleration is constant, we have

6 $$\frac{\,{v}^{2}}{\rho }=constant$$

Therefore,

7 $${v}^{2}\propto \rho \,{\rm{or}}\,{v}^{2}\propto \frac{1}{k}$$

where *k* is the curvature of the trajectory, which is a measure of the sharpness of the turn.

If the bees are holding their centripetal acceleration constant (as hypothesised), then either of the following two (equivalent) predictions must hold:

- a linear relationship between the radius of curvature and speed^2^;
- an inverse relationship between curvature and speed^2^.

**Figure 5:**
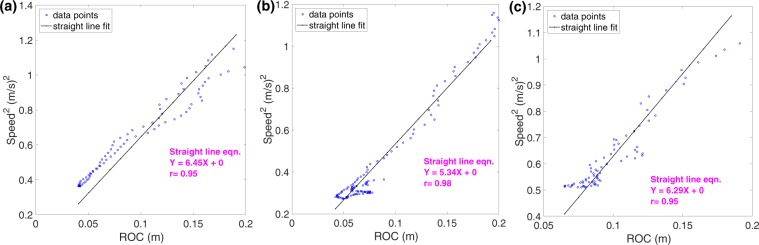
Variation of speed^2^ with radius of curvature (ROC) for the three bees of Fig. 4. The regression line is shown in black in each plot.

To test the hypothesis, we examined the variation of speed^2^ with the radius of curvature (ROC) of the trajectory for individual bees. We plotted the variation of speed^2^ versus ROC for the three example bees illustrated in Fig. 4, which maintained their centripetal acceleration more or less a constant value. These relationships are shown as scatterplots in Fig. 5. This data is plotted for a ROC range of 0.004 m−0.20 m, which corresponds to a curvature magnitude range of 5 m^−1^– 250 m^−1^, as explained at the beginning of this Section. As a result of this windowing process, the trajectories of five bees were removed from the total of 66 bee trajectories. Unless explicitly stated, the number of bees included in all of our subsequent analyses is 61.

For the three scatterplots of speed^2^ versus ROC in Fig. 5, we performed regression analysis on the data by forcing the ‘Y’ intercept to be zero and estimating the slope of the regression line. We used the ‘robust regression’ routine in Matlab, which removes outliers in the data. The correlation efficient values (*r)* computed for the plots in Fig. 5a–c indicate that the regression lines fits the data very well, demonstrating a strong positive, linear correlation between speed^2^ and ROC, as per our prediction. This suggests that each bee is indeed holding the centripetal acceleration constant during the course of its turn. Additional examples from other bees (57 bees in total) are given in Section I (Fig. S1) of the Supplementary Material (SM). The mean value of the slope of the linear regression, computed individually for each of 57 bees, is 2.78 (see Table S1, SM). The correlation coefficients of these linear regressions are consistently high, displaying a mean value of 0.81, with over 85% of the values exceeding 0.70 (see Table S1, SM).

**Figure 6:**
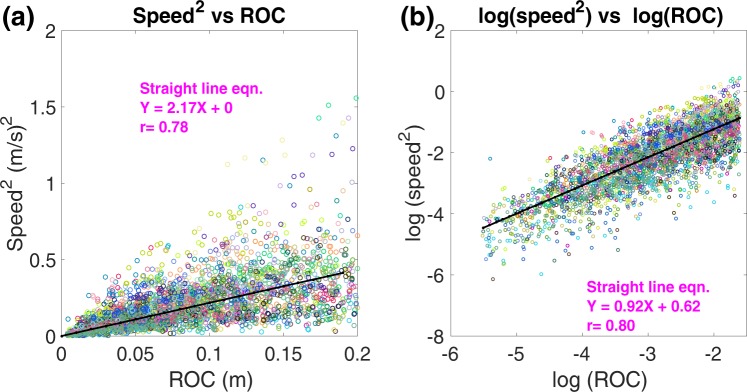
Scatterplot of (**a**) speed^2^ vs radius of curvature (ROC), (**b**) log(speed^2^) vs log(ROC) for 61 bee trajectories.

The relationship between speed^2^ and ROC for all the bees is illustrated in the scatterplot of Fig. 6a. Each colour in the scatterplot represents a different bee. The relatively high degree of variation in this scatterplot is due to the fact that, although each bee tends to show a strong linear correlation between speed^2^ and ROC, the slope of this relationship varies from individual to individual, as can be seen from the plots for individual bees (see Section I of the SM). The overall slope of a linear regression, performed on the data in Fig. 6a, is 2.17. This implies that the magnitude of the centripetal acceleration during a turn, averaged over all the bees, is approximately 2.17 m/s^2^.

To further test our hypothesis, we plotted the log-log relationship between speed^2^ and radius of curvature (ROC). As per our hypothesis, if there exists a linear relationship between speed^2^ and ROC, then the relationship between log(speed^2^) and log(ROC) should be linear, with a slope of 1.0.

Figure 6b shows the relationship between log(speed^2^) and log(ROC), plotted as a scattergram for the data pooled from the 61 bees. This relationship is approximately linear, with a slope of 0.92. This value is close to the value of 1.0 predicted by the hypothesis. The Y-axis intercept of the regression line shown in Fig. 6b is 0.62, from which the average centripetal acceleration can be calculated to be *e*^0.62^ = 1.86 m/s^2^. This is similar to the value of 2.17 m/s^2^ estimated from the slope of the regression of the data in Fig. 6a, the slight difference arising probably because the scatterplot in Fig. 6a is transformed nonlinearly to obtain the scatterplots of Fig. 6b.

Our hypothesis, namely, that bees hold the centrifugal acceleration constant during turns, predicts that at each point in the turn the instantaneous radius of curvature, *ρ*, should be proportional to the square of the instantaneous speed, *v*. Another way to test this hypothesis critically would be to examine whether *ρ* is indeed proportional to the square of *v* – or to the cube of *v*, for example, or some other integer or fractional power of *v*. To do this test, we express equation (5) in a more general form as:

8 $${v}^{n}=c\ast \rho $$

where *n* is the power of *v* and *c* is the constant of proportionality

Taking logarithms on both sides,

$$\mathrm{log}({v}^{n})=\,\mathrm{log}(c\ast \rho )$$

which can be written

9 $$n\,\mathrm{log}(v)=\,\mathrm{log}(c)+\,\mathrm{log}(\rho )$$

or

10 $$\mathrm{log}\,v=\frac{\mathrm{log}(c)}{n}+\frac{\mathrm{log}(\rho )}{n}$$

Thus, the slope of the regression between log(*v*) and log(*ρ*), equal to $\frac{1}{n}$, would allow us to estimate the appropriate value of the power. The y-axis intercept of the regression, equal to $\frac{\mathrm{log}(c)}{n}$, would then enable us to estimate the value of *c*, the coefficient of proportionality.

The scatter plot of log(*v*) vs log(*ρ*) is shown in the SM (Fig. S2). The slope of the regression line is 0.46, yielding a value of 2.2 for *n*. This is very close to the predicted value of 2, additionally supporting our hypothesis of constant centripetal acceleration. The y-axis intercept of the regression line is 0.31. Using *n* = 2.2, we obtain $\mathrm{log}({\rm{c}})=2.2\times 0.31=0.68$, from which we estimate the value of *c* to be 1.97 m/s^2^. This value is similar to those obtained from evaluating the slope of the regressions of the data in Fig. 6a,b (see above).

Thus, broadly speaking, the data of Figs 4–6 and S2 support the hypothesis that bees maintain a more or less constant centripetal acceleration of approximately 2 m/s^2^ during their turns, irrespective of the instantaneous speed or curvature at each point along the turn.

Another way to test our prediction - that the bees are holding their CA constant during their turns - is to re-express equation 6 in terms of the speed, heading rate and CA of the bee as follows:

11 $$\alpha =v\omega $$

or, equivalently,

12 $$v=\alpha \,{\omega }^{-1}$$

where: *α* is the centripetal acceleration in m/s^2^, ω is the heading rate of the bee in rad/s, v is the flight speed of the bee in m/s.

According to equation (12), the heading rate should vary inversely with the speed, if the centripetal acceleration is held constant during the turn. In other words, one would then expect a linear relationship between speed and the reciprocal of the heading rate (heading rate^−1^). The slope of this relationship should represent the magnitude of the centripetal acceleration (*α*). These predictions are analysed and discussed in detail in Section-1 of the SM, under the subheading “Relationship between heading rate and speed”. The interpretation of the results of this analysis is discussed here briefly.

The time course of the variation of the speed and the heading rate are shown for 6 different bees in the left-hand panels of Fig. S3 of the SM. In each case, the heading rate (magenta curve) varies inversely with the speed of the bee (dark curve). This implies that when the bee’s heading rate goes up, its speed decreases in such a way that the variation in CA is small. This observation is supported by the low values of the coefficient of variation of the CA in the six examples (0.14 ± 0.04).

We also verified the constant-CA prediction in a more direct way by plotting the relationship between the instantaneous speed and the reciprocal of the instantaneous heading rate as a scattergram for the 6 examples (Fig. S3, right-hand panels) and performed a linear regression analysis on the data. For each example, the correlation coefficient (r) is greater than 85%, demonstrating a strong positive and linear correlation between the speed and the reciprocal of the heading rate, as per the prediction in equation 12. These findings reinforce our hypothesis that bees hold the centripetal acceleration more or less constant during turns, thereby facilitating coordinated turns.

Table 1 compares the coefficients of variation (CV) of the variables that characterise the trajectories. We observe that, although the CV of the curvature is relatively high, signifying relatively large variations in curvature magnitude, the CV of the centripetal acceleration magnitude is relatively low.

**Table 1:** Mean coefficients of variation (CV) of curvature, speed, speed^2^, and centripetal acceleration magnitude, averaged over 61 bees.

Parameter	Curvature	Speed	speed^2^	Centripetal acceleration magnitude
CV	0.81	0.28	0.54	0.40

This is because the bees are tailoring the flight speed to the curvature in such a way that a potential increase in CA arising from an increase in curvature during the turn is compensated by reducing the speed, and vice-versa, so that the centripetal acceleration is maintained at a more or less constant value through the course of the turn. Thus, the variations in centripetal acceleration during a turn are always low, despite considerable variations in the instantaneous curvature and the speed of the bee. This is evidenced by the relatively low value of CV for the centripetal acceleration, compared to the CVs for the curvature and speed^2^ for all of the 61 bees (see Table 1). In quantitative terms, the centripetal acceleration depends upon the product of (speed^2^) and (curvature), in which both terms display relatively high coefficients of variation (0.54 for speed^2^ and 0.81 for curvature, see Table 1). Despite these high variations, the CV of the product is comparatively low (0.40, Table 1), indicating that changes in the curvature are compensated by changes in speed that are of the appropriate direction and magnitude to ensure that the product (the centripetal acceleration) is held at a more or less constant value.

### Loitering versus close encounter flights

The above analysis includes flight trajectories in which bees make obligatory turns to avoid collisions with other bees, as well as ‘voluntary’ turns while they are loitering in the vicinity of the hive entrance. These can be broadly classified as ‘close encounter’ turns and ‘loitering’ turns. We were interested to compare the characteristics of the two types of turns – one might, for example, expect close encounter turns to be more aggressive, featuring tighter turns and perhaps larger CAs. We distinguished between loitering turns (LTs) and close encounter turns (CETs) by using the following criterion. A bee’s turn was considered to be a LT when there was no other bee within a radius of 100 mm, and a CET when another bee was within a radius of 30 mm. Using this criterion, we classified the turns of 34 bees. In total, there were 77 LTs and 68 CETs. The number of turns executed by each bee is given in Section III (Table S3) of the SM. A comparison of the characteristics of the LTs and the CETs is shown in Table 2 (also see SM Table S2 for data from each individual bee). There are no statistically significant differences between the minimum speed during the turn, the maximum curvature of the turn, or the mean centripetal acceleration during the turn. Thus, LTs and CETs display very similar characteristics.

**Table 2:** Comparison of minimum speed, maximum curvature and mean CA for LTs and CETs of 34 bees.

Parameter	Mean minimum speed (m/s)		Mean maximum curvature (m^−1^)		Mean centripetal acceleration (m/s^2^)	
Average	LT	CET	LT	CET	LT	CET
	0.39	0.34	74.8	73.6	2.88	2.86
*P value (paired sample t-test)*	0.16		0.94		0.87	

### Comparison of characteristics of left and right turns

We were interested to examine whether the bees showed any preferences for turning direction. If the bee’s rotation about its dorsoventral axis (Z_n_) is in the clockwise direction, then the bee turns to the right, and vice versa. In order to determine the turning direction, we computed the 3D rotation vector, which is given by the cross product between the unit velocity vector and the unit centripetal acceleration vector (see Section IV of the SM for explanation). The bee’s turning direction is then obtained by taking the dot product of the 3D rotation vector with the unit vector representing the dorsoventral axis of the bee. If the dot product is positive, the bee is turning right; and vice versa.

**Figure 7:**
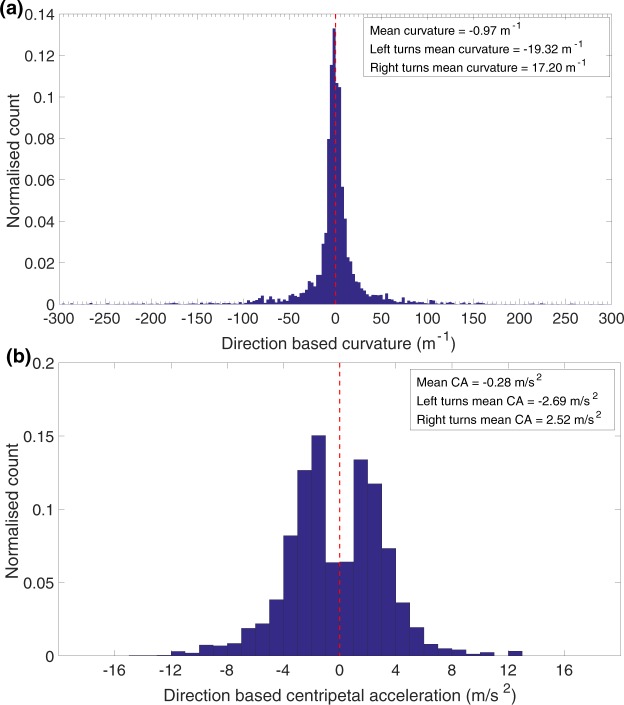
Normalised histogram of (**a**) direction based curvature and (**b**) direction based centripetal acceleration for all bees.

This procedure was used to classify the turning direction, and then to compare the curvatures and centripetal accelerations during left turns with those during right turns. The histogram in Fig. 7a compares the distributions of the curvatures of right turns with those of left turns. Positive curvatures represent right turns, and negative curvatures left turns. The histogram is nearly symmetrical. The mean curvature magnitudes during left (−19.3 m^−1^) and right (17.2 m^−1^) turns are more or less equal and not significantly different (*p* = 0.230; two tailed t-test). The overall mean curvature for all turns (−0.97 m^−1^) is very close to zero and is not significantly different from zero (*p* = 0.277; two tailed t-test). This indicates that turns in either direction are (a) equally likely, and (b) display the same distribution of curvature magnitudes. Thus, the bees flying in our experimental situation do not display any noticeable left-right biases in their turning behaviour.

We also looked for possible biases in the centripetal accelerations associated with left versus right turns. Figure 7b shows a histogram of the distribution of centripetal acceleration for all bees. The peak value of the centripetal acceleration is slightly higher for left turns than for right turns. Apart from this, the histograms for the left and right turns are nearly symmetrical - the mean centripetal acceleration for right turns (+2.5 m/s^2^) is not significantly different from that for the left turns (−2.7 m/s^2^; *p* = 0.460, two tailed t-test). The overall mean centripetal acceleration for all turns (−0.28 m/s^2^) is very close to zero and is not significantly different from zero (*p* = 0.683; two tailed t-test). Thus, as with the curvature magnitudes, there is no major overall bias in the distribution of the centripetal accelerations.

### Body deviation angle analysis of turning bees

From the analysis presented above, we have hypothesized that bees keep their centripetal acceleration almost constant during turns. This strategy might help them perform coordinated turns, without deviating from the intended flight trajectory. Accordingly, we were interested to look for evidence of sideslip. This was done by examining the body deviation angle (BD angle) during turns. We define the BD angle as the angle, measured in the horizontal plane, between the instantaneous flight direction vector and the instantaneous bee’s body orientation vector. This angle is zero when the body axis is aligned with the flight direction. Its polarity is defined to be negative when the body axis points into the turn, and positive when the body axis points away from the turn.

**Figure 8:**
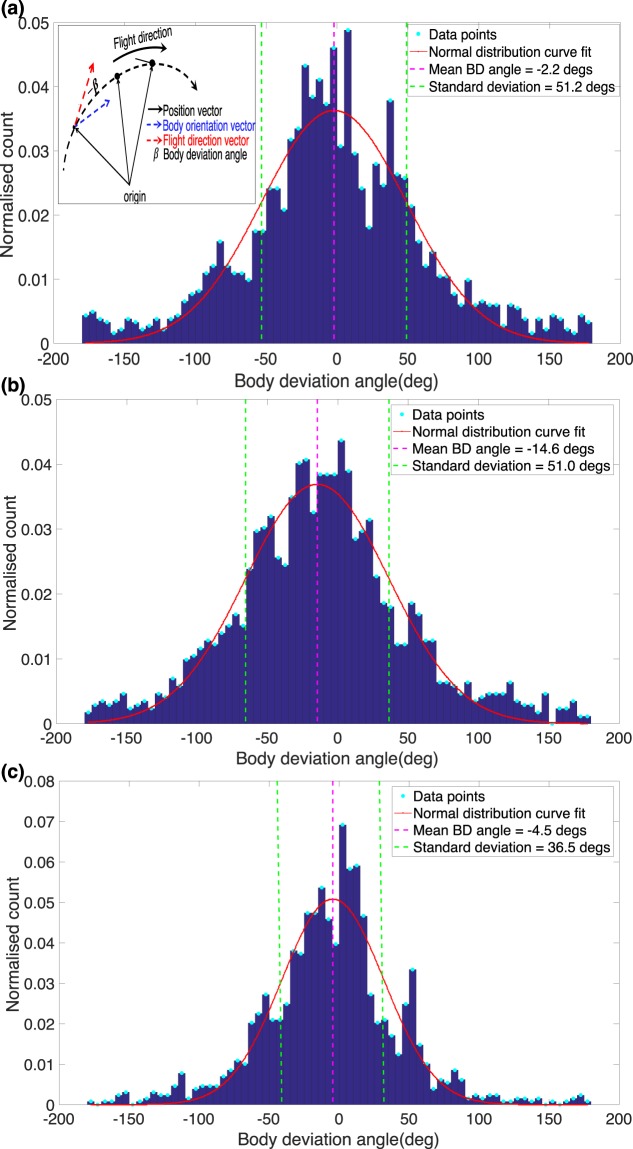
Histogram of BD angles, fitted to a Gaussian distribution during (**a**) left turns, (**b**) right turns and (**c**) straight flights. The inset in panel (**a**) illustrates the parameters involved in estimating the body deviation angle.

We commenced the analysis by calculating the BD angles and plotting their histograms during left turns, right turns and straight flight. “Straight flights” were defined to be sections of the trajectory in which the curvature magnitude was lower than a threshold of 5.0, and turning flights were sections in which the curvature magnitude exceeded 5.0, with the polarity of the curvature defining the direction of the turn. The results are shown in Fig. 8, where each histogram has been fitted to a Gaussian distribution.

The mean and standard deviation of the body deviation angle after correction for estimated errors in the measurement of the direction of body orientation and flight direction from the video images, are given in Section V of the SM. The results (see SM Table S4) reveal that the BD histograms for left turns, right turns and straight flight display a mean value close to zero, but a broad standard deviation of about 50 deg. This implies that, although the body orientation can occasionally deviate substantially from the direction of flight, the deviations are more or less symmetrical, with roughly half of the deviations pointing into the turn and the other half pointing outward. This is true for all three conditions - left turns, right turns, and even in straight flight. This suggests that the observed BDs are not a reflection of uncontrolled turns that involve sideslips; rather, they are a natural characteristic of the loitering bees, in which the body does not point consistently in the flight direction. Sideslips, if present, would be reflected in the left and right-turn histograms by an increased frequency of negative BD angles (body pointing into the turn) - which is not the case. Instances where the magnitude of the BD angle exceeds 90 deg represent situations where the bee is moving temporarily backwards. In this condition, the BD angles are again positive or negative equally frequently.

**Figure 9:**
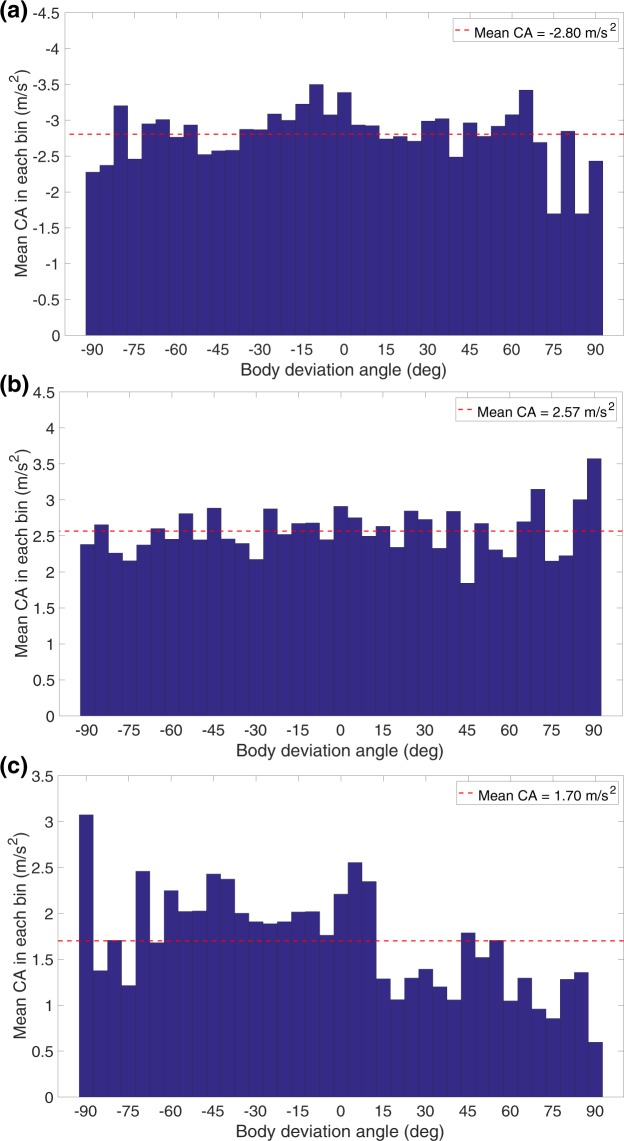
Variation of mean centripetal acceleration magnitude with BD angle during (**a**) left turns, (**b**) right turns and **(c**) straight flights. The dashed red line represents the overall mean in each case.

To further explore the existence of sideslips, we computed the mean value of the magnitude of the centripetal acceleration in each bin of the body deviation angle histograms of Fig. 8. The results, shown in Fig. 9, indicate that the magnitude of the centripetal acceleration is more or less constant, independent of body deviation angle. This is true for right turns, left turns, and straight flights. BD angles greater than 90 deg are not included in the histograms of Fig. 9, since those instances are not turning flights, but rather situations in which the bee is moving temporarily backwards.

The mean value of the CA magnitude, computed from the histograms of Fig. 9, are −2.80 m/s^2^ for left turns, 2.57 m/s^2^ for right turns, and 1.70 m/s^2^ for close-to-straight flights. Secondly, the observation that the CA magnitudes are similar even at large negative and positive values of BD angle (i.e. irrespective of whether the body is pointing sharply into or away from the turn), makes it very unlikely that the large negative values of BD angles (when the body is pointing sharply into the turn) are associated with uncontrolled sideslips or skids. In summary, the data in Figs 8 and 9 and Table S4 suggest that the bees flying in the cloud are never overcome by the centrifugal forces that are encountered while executing these turns, which would result in uncontrolled sideslips.

## Discussion

We have investigated the turning flight characteristics of loitering honeybees in a semi-outdoor environment comprising a number of bees flying in close proximity to each other, trying to enter a blocked hive. We commenced our analysis by studying how the kinematics of bees vary in a cloud. In general, the speed of the bee varies continuously through its flight path.

Bomphrey *et al*.^10^ measured the characteristics of the flight envelope of freely flying blowflies (*Calliphora vicinia*) in an ingeniously designed ‘corner cube’ arena, which enabled them to film and reconstruct the flies’ 3D trajectories using a single video camera. Their results, compared to ours, indicate that blowflies display flight manoeuvres that are generally more aggressive than those of honeybees in similar conditions, featuring higher mean tangential and centripetal accelerations, but shallower turns. However, their study did not explore the relationships between these variables during turning behaviour.

Our study, which focuses on turning behaviour, shows that the flight speed tends to decrease whilst entering a turn, and increase whilst exiting it. This general pattern of speed variation has been documented in a number of other aerial and terrestrial animal species, for example fruit flies^5^, bats^6^, horses^2^ and northern quolls^3^. However, none of these studies have quantitatively examined the relationship between speed and turning radius. Our study does this and finds that, during the course of a turn, flight speed varies with curvature in such a way that the centrifugal force is maintained at a more or less constant value, irrespective of the moment-to-moment variations in speed and curvature.

Our results also provide an estimate of this centrifugal force. The histogram of Fig. 7b indicates that the mean centripetal acceleration is −2.69 m/s^2^ during left turns, and 2.52 m/s^2^ during right turns. This is in good agreement with the data from Fig. 3d (2.80 m/s^2^), and from Fig. 9a,b, which indicate mean centripetal accelerations of −2.80 m/s^2^ for left turns, and 2.57 m/s^2^ for right turns. It is also in good agreement with the mean value of 2.78 m/s^2^ obtained from the individual slopes of the speed^2^ vs ROC regressions for 57 bees (Table S1, SM). All of these numbers are consistently slightly higher than those inferred from the analyses of the scatterplots of Fig. 6a (2.17 m/s^2^), Fig. 6b (1.86 m/s^2^) and Fig. S2 (1.97 m/s^2^). We believe that the reason for this slight discrepancy is that, in the scatterplots, data from the bees were pooled without accounting for the flight duration of each bee, which would mean that bees that flew longer trajectories would have made a greater contribution to the estimated parameters. Therefore, it is likely that the values obtained from Figs 3d, 7b, 9a,b and Table S1 are most representative of the true mean magnitude of the centripetal acceleration. The grand mean of these mean values is 2.69 m/s^2^, which is about 27% of the acceleration due to gravity (9.81 m/s^2^). This means that the average centrifugal force experienced by a turning bee is approximately 30% of the bee’s weight, which we propose is low enough to permit coordinated turns without incurring unwanted sideslips. Orchestrating turns in this way would ensure that the insect is never overcome by the centrifugal force during the turn, and always maintains the intended (curved) trajectory.

To probe our hypothesis further, we examined whether bees undergo sideslips during turns. If a bee is unable to resist the centrifugal force that it experiences during a turn, we would expect its body to point into the turn – analogous to a car that skids out of control while making a sharp turn. Our findings (Fig. 8) indicate that there is no systematic bias in the body deviation angle that is correlated with the direction of the turn – in other words, there is no evidence that the body points preferentially into the turn. Moreover, the finding that the width of the body deviation histogram is approximately the same for left turns, right turns and nearly straight flights (see SM Table S4), suggests that the variations in the body deviation angle are a normal feature of honeybee flight in these experimental conditions, and do not reflect sideslips. Additional evidence for the lack of sideslips comes from the plots of centripetal acceleration versus body deviation angle (Fig. 9), which reveal that the centripetal acceleration is roughly constant – it does not vary with the body deviation angle. If sideslips were to occur, one would expect large body deviations into the turn (negative body deviation angles) to be associated with larger centripetal accelerations. This is clearly not the case – there is no correlation between the body deviation angle and the centripetal acceleration (or, equivalently, centrifugal force) – which, again, suggests that the observed variation in the body deviation angles is not due to the presence of uncontrolled turns. Our data of course includes several instances of turning bees in which the axis of the body is not aligned with the flight direction – as is evidenced by the broad histograms in Fig. 8. However, such flight segments, where the bee’s translational motion contains a lateral component, are likely to be controlled lateral motions, rather than uncontrolled sideslips resulting from a capitulation to the centrifugal force.

Our observation that turning bees hold the centripetal acceleration constant is further supported by the finding that the magnitude of this acceleration is practically the same during loitering turns and turns that involve a close encounter with another bee. Thus, it appears bees flying in a cloud display the same turning dynamics, regardless of the context in which the turn occurs. Further investigation is currently under way to explore the nature of the sensory information that is used to guide collision avoidance manoeuvres during these close encounter turns.

Finally, our study also indicates that, under our experimental conditions, left and right turns display similar characteristics, when the data are pooled across the bees that were investigated. Thus, it appears that, as a whole, the group of bees flying in our bee cloud does not exhibit a preferred turning direction (left or right) -although we cannot rule out the possibility that individual bees have turning biases, which would be a topic for future investigation. On the other hand, army ants, fish^11^ and bats^12^ rotate in a particular direction displaying a collective turning behaviour that could promote collision avoidance. In summary, our study documents a turning strategy that is used by honeybees to execute controlled, side-slip free turns while they are in a loitering mode of flight in a bee cloud. It would be interesting to examine whether this strategy also applies to flight in other conditions.

## Electronic supplementary material

Supplementary Information

## Data Availability

The datasets generated during the current study are available from the corresponding author on reasonable request.
