# Author Correction: Coordinated Turning Behaviour of Loitering Honeybees

**DOI:** 10.1038/s41598-019-41874-y

**Published:** 2019-05-24

**Authors:** Mandiyam Y. Mahadeeswara, Mandyam V. Srinivasan

**Affiliations:** 1 Queensland Brain Institute, University of Queensland, St Lucia, QLD Australia; 2 School of Information Technology and Electrical Engineering, University of Queensland, St Lucia, QLD Australia

Correction to: *Scientific Reports* 10.1038/s41598-018-35307-5, published online 16 November 2018

This Article contains an error in Equation 2.

$${a}_{N}=\frac{||{\boldsymbol{z}}^{\prime} ({\boldsymbol{t}})\times {\boldsymbol{r}}^{\prime\prime} ({\boldsymbol{t}})||}{||{\boldsymbol{r}}^{\prime} ({\boldsymbol{t}})||}$$

should read:

$${a}_{N}=\frac{||{\boldsymbol{r}}^{\prime} ({\boldsymbol{t}})\times {\boldsymbol{r}}^{\prime\prime} ({\boldsymbol{t}})||}{||{\boldsymbol{r}}^{\prime} ({\boldsymbol{t}})||}$$

