# Supplementary material for: Coordinated Turning Behaviour of Loitering Honeybees
Source: Sci Rep. 2018 Nov 16;8:16942. doi: 10.1038/s41598-018-35307-5 (PMC6240102; doi:10.1038/s41598-018-35307-5)
Supplement: Supplementary file 1 — Supplementary Information [file 41598_2018_35307_MOESM1_ESM.pdf]

SUPPLEMENTARY MATERIAL

COORDINATED TURNING BEHAVIOUR OF LOITERING HONEYBEES

Mandiyam Y. Mahadeeswara[1] and Mandyam V. Srinivasan[1,2]

SECTION I

Scatterplots of speed² versus radius of curvature and curvature

Fig S1 (below) complements the data of Fig 5 in the main text by showing scatterplots of speed^2 versus radius of curvature, and speed^2 versus curvature for 7 additional bees. These scatterplots show approximately linear and inverse relationships respectively, as per our prediction.

Fig S1. *Variation of speed² with radius of curvature (ROC) and curvature for 7 individual bees. (This figure spans pages 1-3).*

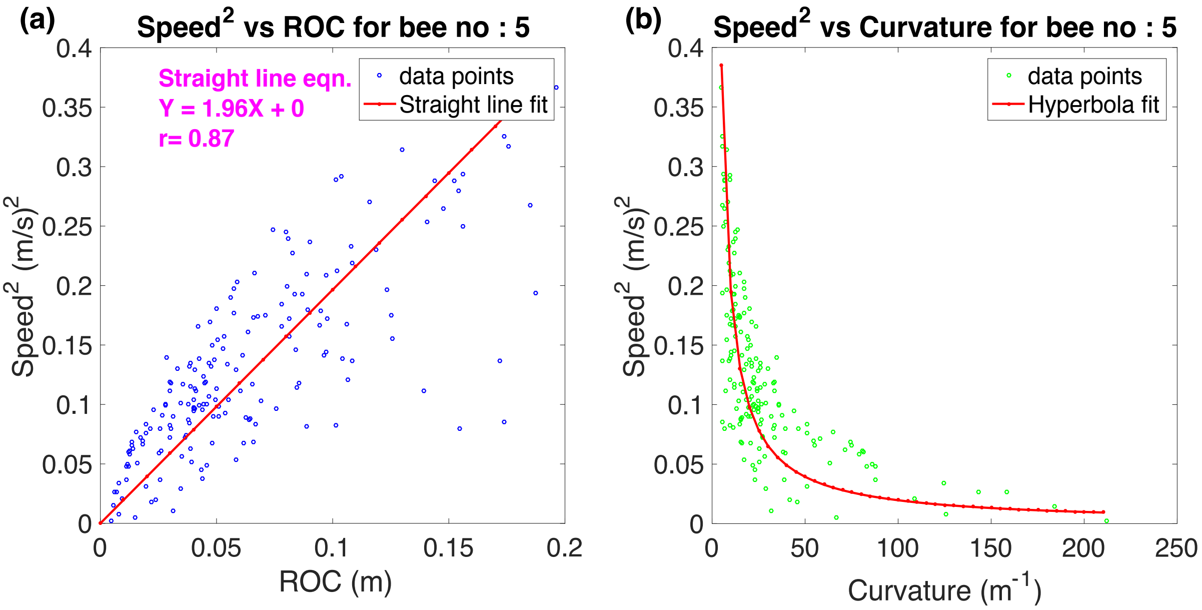

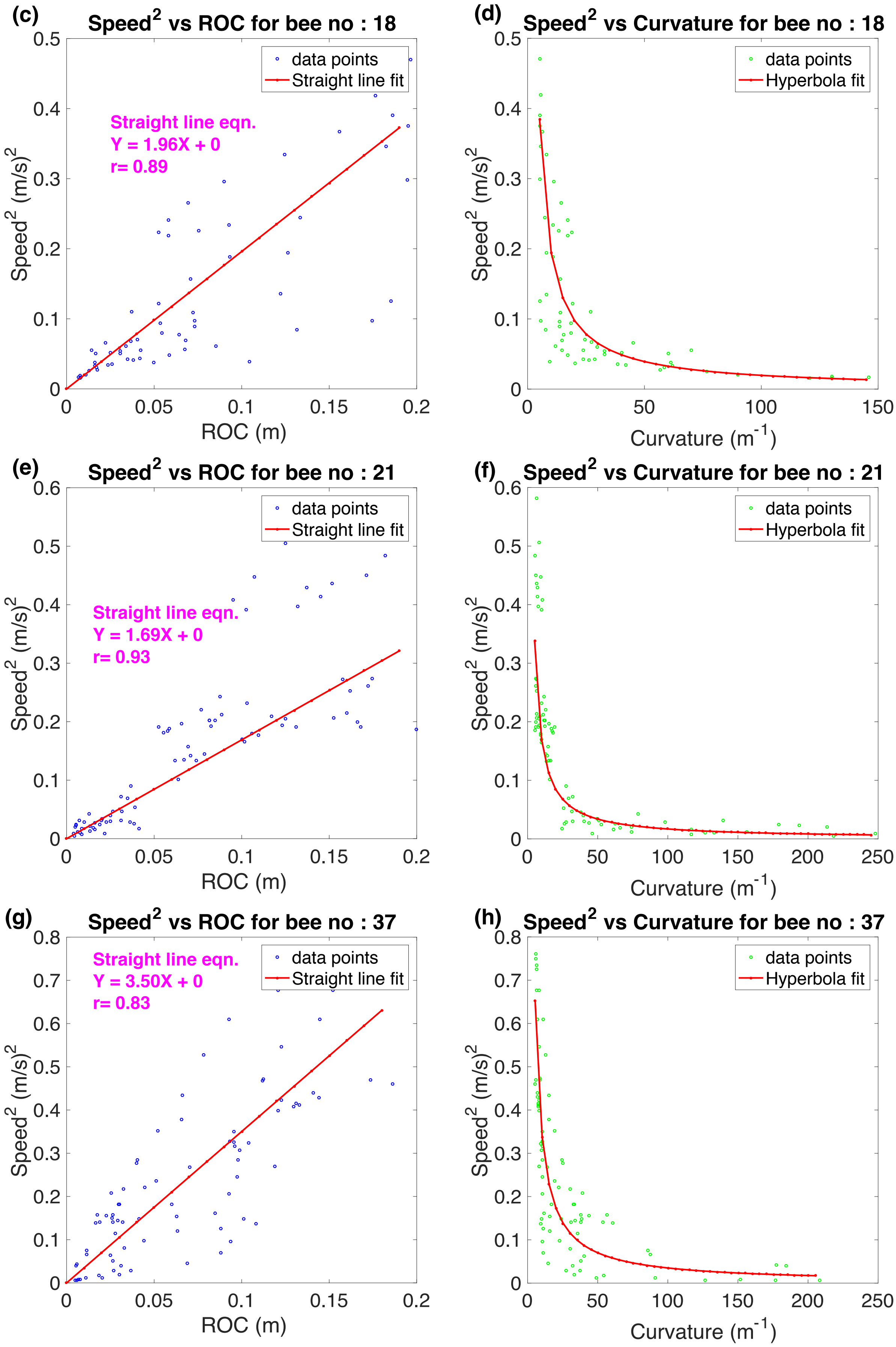
(c)
Speed2 vs ROC for bee no : 18
data points
Straight line fit
Straight line eqn.
Y = 1.96X + 0
r= 0.89
Speed2 (m/s)2
ROC (m)
(d)
Speed2 vs Curvature for bee no : 18
data points
Hyperbola fit
Speed2 (m/s)2
Curvature (m-1)
(e)
Speed2 vs ROC for bee no : 21
data points
Straight line fit
Straight line eqn.
Y = 1.69X + 0
r= 0.93
Speed2 (m/s)2
ROC (m)
(f)
Speed2 vs Curvature for bee no : 21
data points
Hyperbola fit
Speed2 (m/s)2
Curvature (m-1)
(g)
Speed2 vs ROC for bee no : 37
Straight line eqn.
Y = 3.50X + 0
r= 0.83
data points
Straight line fit
Speed2 (m/s)2
ROC (m)
(h)
Speed2 vs Curvature for bee no : 37
data points
Hyperbola fit
Speed2 (m/s)2
Curvature (m-1)

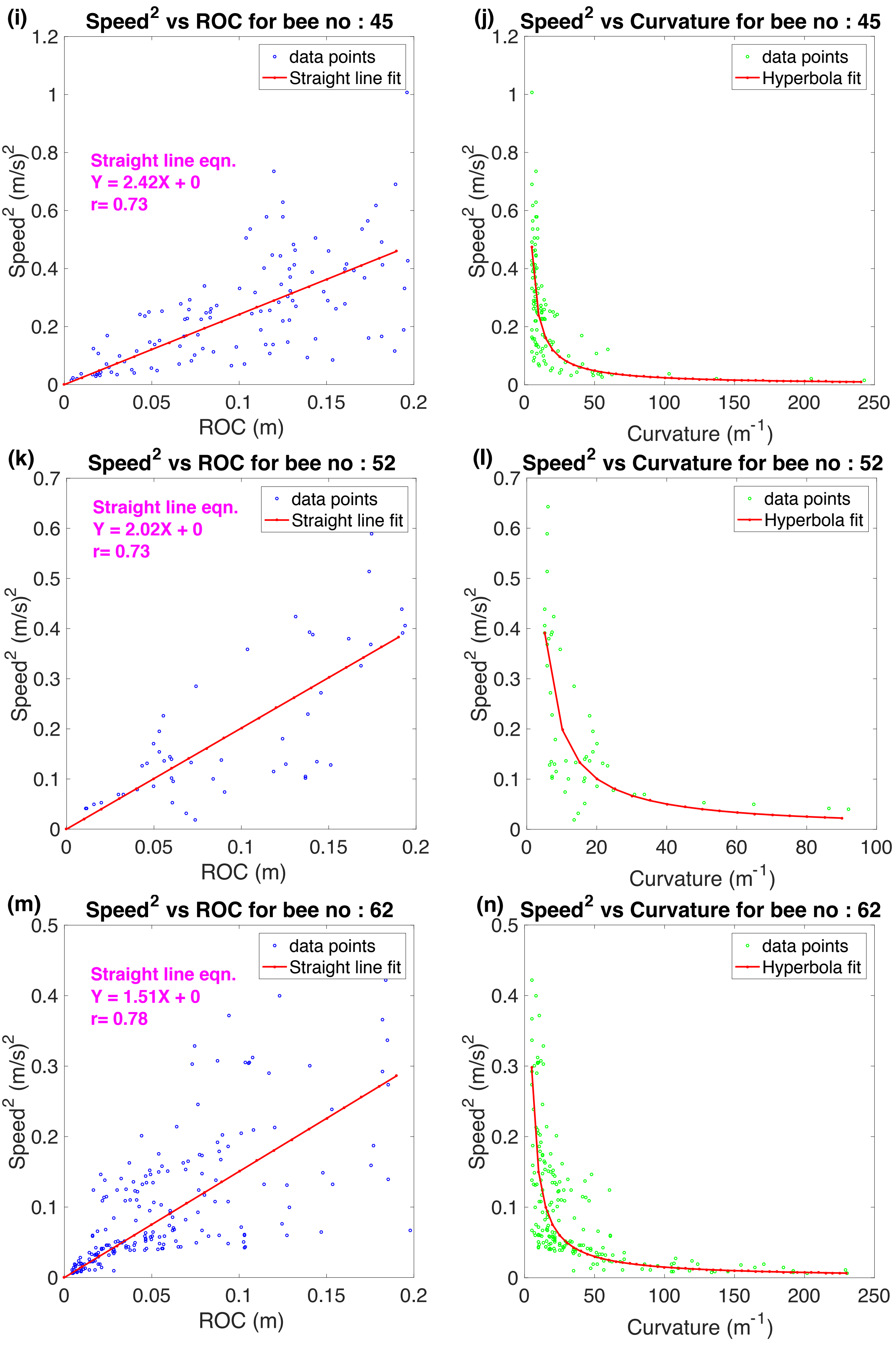

(i)
Speed^2 vs ROC for bee no : 45
data points
Straight line fit
Straight line eqn.
Y = 2.42X + 0
r= 0.73
Speed^2 (m/s)^2
ROC (m)
(j)
Speed^2 vs Curvature for bee no : 45
data points
Hyperbola fit
Speed^2 (m/s)^2
Curvature (m^{-1})
(k)
Speed^2 vs ROC for bee no : 52
Straight line eqn.
Y = 2.02X + 0
r= 0.73
data points
Straight line fit
Speed^2 (m/s)^2
ROC (m)
(l)
Speed^2 vs Curvature for bee no : 52
data points
Hyperbola fit
Speed^2 (m/s)^2
Curvature (m^{-1})
(m)
Speed^2 vs ROC for bee no : 62
data points
Straight line fit
Straight line eqn.
Y = 1.51X + 0
r= 0.78
Speed^2 (m/s)^2
ROC (m)
(n)
Speed^2 vs Curvature for bee no : 62
data points
Hyperbola fit
Speed^2 (m/s)^2
Curvature (m^{-1})

Relationship between log(speed) and log(ROC)

Fig S2 (below) shows the relationship between log(speed) and log (ROC) for all 61 bees. The value of CA estimated from this scatterplot is approximately equal to the value of slope obtained from the regression analysis on the data in Fig 6a. This supports our prediction that the ROC is proportional to speed^2, and not any other power of speed.

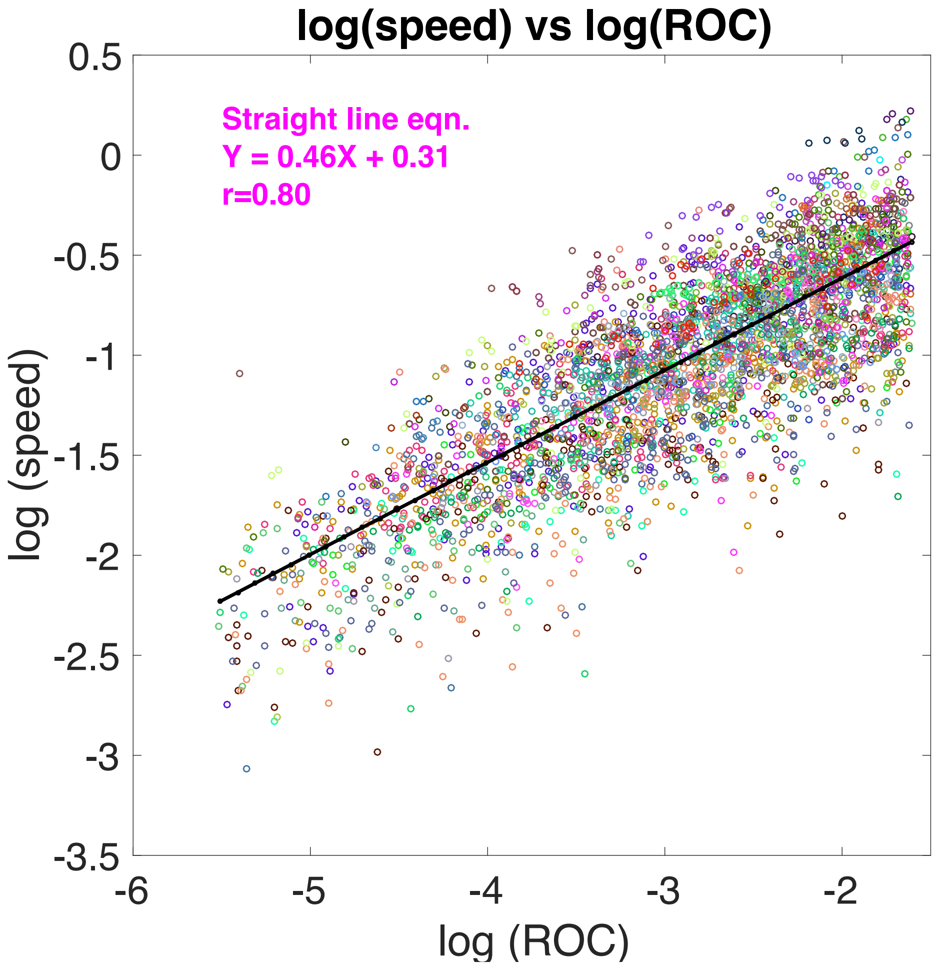

Fig S2. *Variation of log(speed) versus log(ROC) for all 61 bee trajectories.*

Relationship between heading rate and speed

If the centripetal acceleration (α) is to remain constant (as hypothesised) during a turn, then equation (6) can be written as:

$$\frac{v^2}{\rho} = \alpha \tag{S1}$$

We also know from basic kinematics that the rate of change of heading, ω (rad/s) is related to the flight speed 'v' and the turning radius 'ρ' by

$$v = \rho\omega \tag{S2}$$

Combining (S1) and (S2), we may write

$$v = \frac{\alpha}{\omega} \tag{S3}$$

Or, equivalently,

$$v = \alpha\, \omega^{-1} \tag{S4}$$

Our hypothesis now becomes:

If the centripetal acceleration is held constant, then the speed (v) would be inversely proportional to the rate of change of heading (ω); or, equivalently, proportional to (ω^{-1}).

In order to test our hypothesis, we examined the variation of heading rate with speed for the same example bees shown in Fig 4. The corresponding plots are shown in Fig S3(a-c). We have also analysed data for three additional new bees, which are also included in the same Fig S3(d-f).

As the bee enters the turn, the speed (dark curve, left-hand panel) decreases gradually, while the heading rate (magenta curve, left-hand panel) increases concomitantly. Beyond the halfway point of the turn, the flight speed increases, with a corresponding decrease in the heading rate.

There is thus an inverse relationship between the heading rate and speed of the bee, as predicted by equation (S4). As a consequence of this inverse relationship, the variation in the CA would be expected to be low. This is confirmed by the low values of coefficient of variation of CA displayed by all 6 bees (0.16, 0.13, 0.09, 0.11, 0.12 and 0.20).

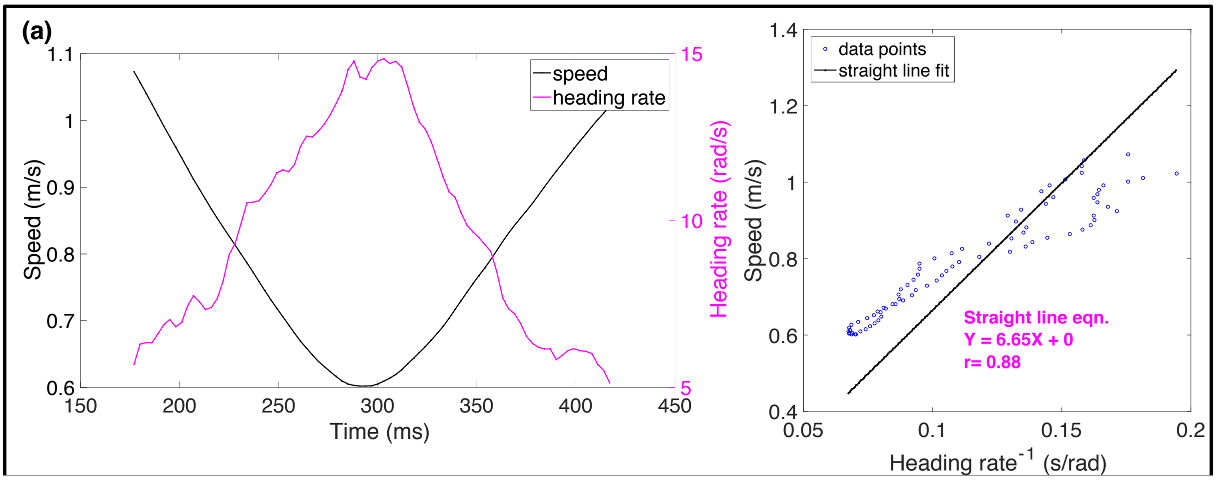

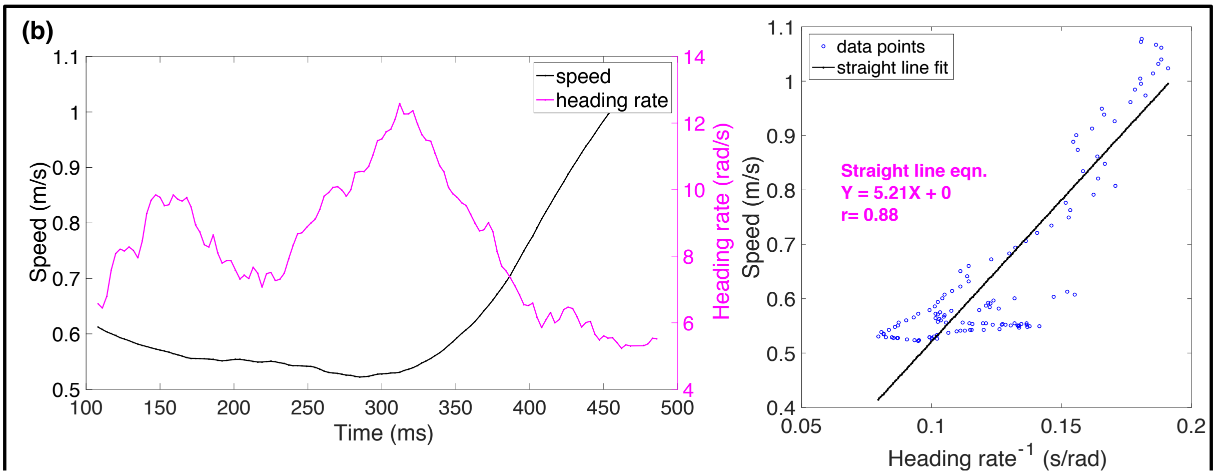

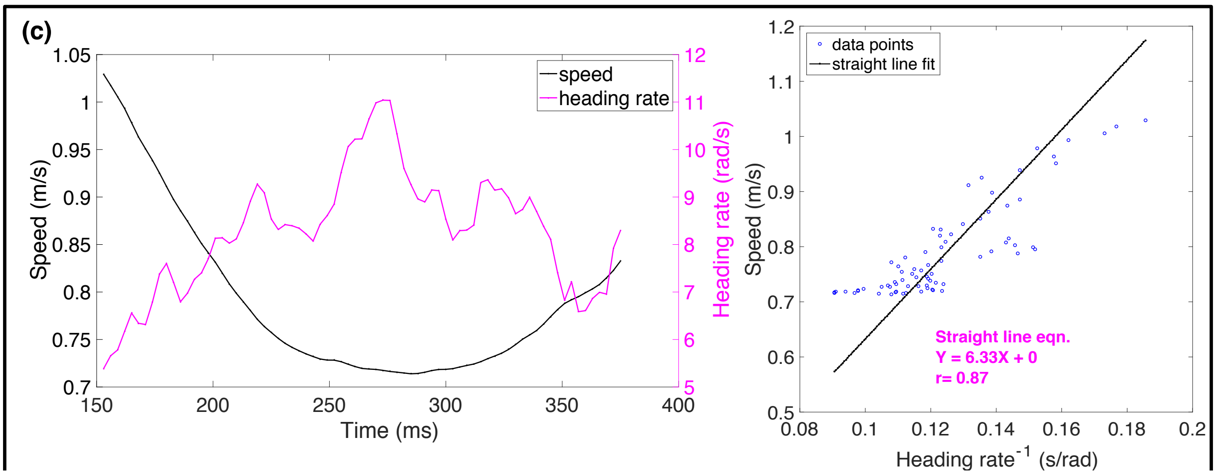

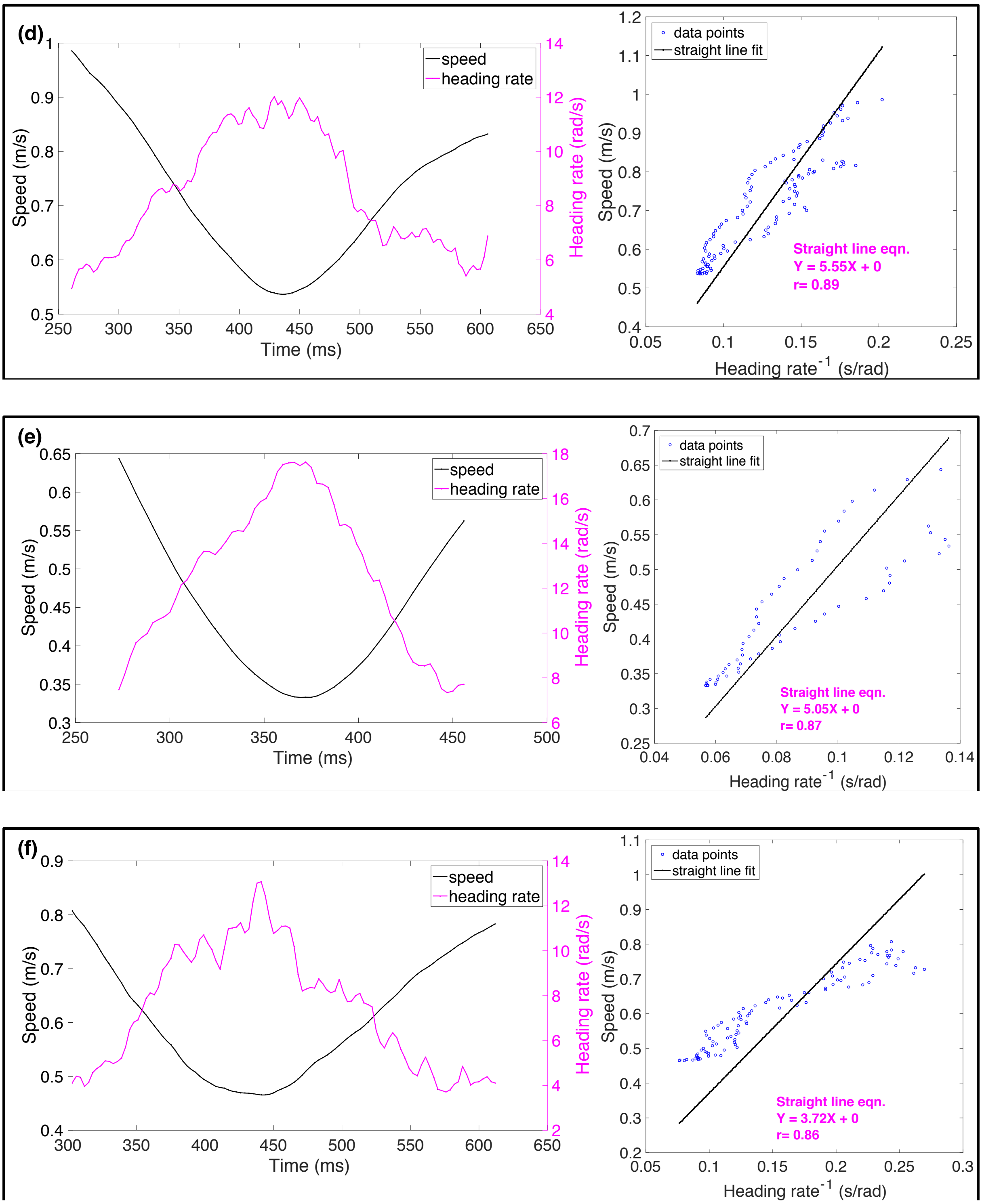

Fig S3. *(a-f) Time course of speed and heading rate for 6 different bees, along with the scattergrams showing the corresponding relationship between speed and inverse of heading rate. The left-hand panels show the variation of speed (left ordinate; dark curve) and heading rate (right ordinate; magenta curve) as a function of time (abscissa). The right-hand panels show the relationship between speed and heading rate*$^{-1}$ *for the 6 bees.*

We validated the prediction more quantitatively by estimating the correlation coefficient of a regression line fitted to the scatterplots in the right hand panels of Fig S3. The estimated correlation coefficients (0.88, 0.88, 0.87, 0.89, 0.87 and 0.86) indicate a strong linear relationship between the speed and the reciprocal of the heading rate, as predicted by equation (S4). This data again strongly supports our hypothesis that the CA is held constant during turns.

The values of centripetal acceleration, inferred from the slopes of the regressions in Fig S3, are almost equal to those inferred from the speed^2 vs ROC plots shown for the three example bees in Fig 5. This exercise demonstrates the validity of the constant-CA hypothesis, even when it is examined by using a different set of dynamic variables.

The inferred values of CA shown in Fig S3 are larger than those shown in Fig S1. One reason for this might be that, while bees tend to hold the value of the CA constant during the course of a turn, the preferred CA could vary from one individual to another – as is suggested by the large spread of slopes of the speed^2 vs ROC relationships in Fig 6a. Another reason may be that the scatterplots of Fig S3 show data for a single turn, while those of Fig S1 pertain to the entire flight trajectory, which is likely to comprise turns as well as flight segments that are nearly straight (straight flight would generate zero CA) . In any case, the positive and strong linear correlation between speed^2 and ROC (Fig. S1), and between speed and the reciprocal of the turning rate (Fig S3) indicate that the bees are holding the CA constant during turns.

SECTION II

Table S1. *Slope and correlation coefficient of (speed^2) vs (radius of curvature) obtained from scatterplots for individual bees.*
NA – Bees (15,16, 40, 55, 58) did not meet the curvature constraint, and were therefore not considered in the analysis.
LS –The trajectories of bees (17, 36, 39 and 49) were reduced to just 3 points as a result of applying the curvature constraint, and were therefore not analysed.

Bee number	***Slope of (Speed^2) vs (Radius of curvature) m/s^2***	***Correlation coefficient (r)***
1	2.71	0.52
2	2.51	0.80
3	1.14	0.99
4	1.61	0.99
5	1.97	0.87
6	1.19	0.84
7	4.40	0.60
8	3.99	0.62
9	1.39	0.88
10	2.81	0.91
11	1.71	0.86
12	2.10	0.87
13	1.72	0.87
14	2.65	0.71
15	NA	NA
16	NA	NA
17	LS	LS
18	1.96	0.89
19	2.37	0.87
20	1.03	0.88
21	1.69	0.93
22	2.61	0.89
23	1.99	0.84
24	1.14	0.76
25	2.40	0.64
26	1.74	0.82
27	2.04	0.64
28	4.30	0.81
29	2.43	0.73
30	2.75	0.80
31	4.95	0.37
32	3.62	0.95
33	2.35	0.75
34	1.38	0.79
35	2.89	0.89

36	*LS*	*LS*
37	*3.50*	*0.83*
38	*5.25*	*0.89*
39	*LS*	*LS*
40	*NA*	*NA*
41	*2.99*	*0.78*
42	*5.44*	*0.97*
43	*1.18*	*0.81*
44	*3.55*	*0.84*
45	*2.42*	*0.73*
46	*1.78*	*0.81*
47	*1.88*	*0.77*
48	*1.78*	*0.79*
49	*LS*	*LS*
50	*8.29*	*0.82*
51	*2.58*	*0.91*
52	*2.02*	*0.73*
53	*4.27*	*0.82*
54	*4.46*	*0.85*
55	*NA*	*NA*
56	*2.34*	*0.90*
57	*2.76*	*0.76*
58	*NA*	*NA*
59	*6.78*	*0.91*
60	*2.87*	*0.94*
61	*2.05*	*0.85*
62	*1.51*	*0.78*
63	*2.62*	*0.78*
64	*1.51*	*0.81*
65	*2.84*	*0.96*
66	*6.31*	*0.57*
MEAN	***2.78***	***0.81***

SECTION III

Table S2. *Centripetal acceleration, minimum speed and maximum curvature of 34 bees during loitering turns (LT) and close encounter turns (CET).*

Bee number	***Centripetal acceleration(m/s²)***		***Minimum speed (m/s)***		***Maximum curvature (m⁻¹)***	
	LT	***CET***	***LT***	***CET***	***LT***	***CET***
1	*3.34*	*2.45*	*0.10*	*0.22*	*166.7*	*141.9*
2	*2.85*	*2.37*	*0.27*	*0.22*	*57.9*	*98.3*
3	*3.01*	*1.76*	*0.13*	*0.17*	*218.4*	*84.6*
4	*1.42*	*1.04*	*0.40*	*0.10*	*10.9*	*215.5*
5	*2.86*	*2.85*	*0.65*	*0.33*	*10.5*	*44.2*
6	*2.27*	*2.13*	*0.13*	*0.18*	*203.9*	*101.6*
7	*3.49*	*2.69*	*0.37*	*0.26*	*41.4*	*59.2*
8	*4.76*	*4.76*	*1.00*	*1.00*	*4.9*	*4.9*
9	*3.54*	*3.68*	*0.89*	*0.87*	*5.3*	*5.3*
10	*2.11*	*2.52*	*0.58*	*0.28*	*9.1*	*36.0*
11	*1.83*	*1.90*	*0.13*	*0.14*	*194.8*	*137.1*
12	*2.97*	*1.53*	*0.44*	*0.09*	*19.0*	*247.9*
13	*2.69*	*2.27*	*0.36*	*0.27*	*19.0*	*50.3*
14	*3.36*	*1.86*	*0.20*	*0.21*	*73.4*	*93.1*
15	*3.05*	*3.39*	*0.24*	*0.21*	*121.6*	*80.1*
16	*5.68*	*6.32*	*0.52*	*0.34*	*30.7*	*220.8*
17	*2.94*	*1.78*	*0.37*	*0.23*	*19.6*	*84.8*
18	*4.28*	*4.39*	*0.26*	*0.26*	*82.0*	*82.0*
19	*1.44*	*2.03*	*0.47*	*0.46*	*7.4*	*9.9*
20	*1.40*	*1.80*	*0.29*	*0.20*	*23.0*	*62.3*
21	*3.39*	*4.37*	*0.18*	*0.35*	*136.0*	*52.5*
22	*1.67*	*1.26*	*0.12*	*0.37*	*199.7*	*12.6*
23	*2.04*	*3.41*	*0.80*	*0.57*	*4.1*	*16.5*
24	*2.37*	*3.51*	*0.13*	*0.67*	*243.0*	*9.6*
25	*1.55*	*2.18*	*0.50*	*0.24*	*7.1*	*50.0*
26	*2.99*	*1.86*	*0.43*	*0.32*	*27.6*	*37.6*
27	*2.45*	*2.06*	*0.16*	*0.25*	*144.4*	*81.9*
28	*1.24*	*2.30*	*0.29*	*0.27*	*20.0*	*19.8*
29	*5.96*	*6.57*	*0.49*	*0.49*	*41.0*	*41.0*
30	*6.90*	*6.94*	*0.74*	*0.74*	*18.2*	*18.2*
31	*1.92*	*2.11*	*0.08*	*0.17*	*230.2*	*159.0*
32	*2.22*	*2.44*	*0.46*	*0.48*	*12.4*	*15.2*
33	*2.12*	*1.46*	*0.17*	*0.28*	*135.3*	*23.5*
34	*1.96*	*3.28*	*0.79*	*0.23*	*3.2*	*102.6*
Mean	***2.88***	***2.86***	***0.39***	***0.34***	***74.8***	***73.6***
P value	***0.87***		***0.16***		***0.94***	

Table S3. *Number of turns executed by each bee during loitering turns (LT) and close encounter turns (CET).*

Bee number	***Number of turns***	
	LT	***CET***
1	*5*	*5*
2	*3*	*7*
3	*3*	*4*
4	*1*	*1*
5	*1*	*2*
6	*4*	*3*
7	*5*	*3*
8	*1*	*1*
9	*1*	*1*
10	*1*	*3*
11	*1*	*2*
12	*1*	*1*
13	*5*	*2*
14	*3*	*3*
15	*1*	*1*
16	*2*	*1*
17	*3*	*3*
18	*1*	*1*
19	*1*	*1*
20	*2*	*2*
21	*2*	*2*
22	*2*	*2*
23	*2*	*1*
24	*4*	*1*
25	*2*	*1*
26	*2*	*2*
27	*2*	*2*
28	*2*	*2*
29	*2*	*1*
30	*1*	*1*
31	*5*	*3*
32	*3*	*1*
33	*2*	*1*
34	*1*	*1*
Total	*77*	***68***

SECTION IV

Computation of direction based centripetal acceleration

The direction of a bee's turn about its dorsoventral axis can be obtained by computing the direction of its 3D rotation vector and projecting this vector on the dorsoventral axis of the bee, which we denote by Z_n. If the rotation about Z_n is in the clockwise direction the bee turns to the right, and vice-versa. The procedure involves 2 major steps. Firstly, we compute the 3D rotation vector as the cross product between the unit forward velocity vector and the unit centripetal acceleration vector. This 3D rotation vector is then projected onto the bee's dorsoventral axis (Z_n) to obtain the direction of the bees' turn (right or left), according to whether the result is positive or negative.

Denoting the unit centripetal acceleration vector by C_n and the unit velocity vector by V_n, the 3D rotation vector D_n is given by:

$$D_n = V_n \times C_n$$

The second step is to construct the bee's coordinate axes. Using the bee's head to tail vector, we first define an axis that points in the direction of the bee's longitudinal body axis. This axis is denoted by the unit body vector X_n.

The lateral axis (Y axis) of the bee's body (Y_n) can be written as:

$$Y_n = X_n \times Z$$

where 'Z' is the unit vector directed along the vertical axis of the world co-ordinate system, and ′ × ' denotes the cross product. We then compute the vector Z_n, which is the direction of the dorsal axis of the bee, as the cross product between Y_n and X_n :

$$Z_n = X_n \times Y_n$$

Finally, by projecting the 3D rotation vector(D_n) on to Zn, we obtain a scalar DC_n whose polarity defines the direction of rotation of the bee about its dorsoventral axis:

$$DC_n = D_n \,.\, Z_n$$

where (.) denotes the dot product of the two vectors.

The polarity of DC_n gives the direction of the turn: A positive value signifies a turn to the right, and a negative value a turn to the left.

SECTION V

Correction of raw measurements of BD angle

The raw measurements of the BD angle do not account for manual digitisation errors present in the data. Based on the average pixel length (10) and width (2) of the bees in the video images, and an average movement of 10 pixels between frames, we estimate the standard deviation of the error in the measurement of the body orientation and the flight direction to each be approximately 12 deg, on average. Therefore, the standard deviation of the error $\sigma_{BDerror}$ in the measurement of the BD angle (which is the difference between the two measured directions) is $\sigma_{BDerror} = \sqrt{2}(12) \approx 17\ deg$. The measured standard deviation $\sigma_{BDmeasured}$ of the BD angle can then be corrected to obtain the corrected standard deviation $\sigma_{BDcorrected}$ from

$$\sigma_{BDcorrected} = \sqrt{{\sigma_{BDmeasured}}^2 - {\sigma_{BDerror}}^2}$$

The corrected values are given in the fourth column of Table S4.

Table S4. *Mean, raw standard deviation and corrected standard deviation of BD angle*

Flight category	Mean BD angle (deg)	Raw standard deviation of BD angle (deg)	Corrected standard deviation of BD angle (deg)
Left turns	-2.2	51.2	48.2
Right turns	-14.6	51.0	48.0
Straight flights	-4.5	36.5	32.3
